# A student-led course in clinical reasoning in the core curriculum

**DOI:** 10.5116/ijme.4c18.94a5

**Published:** 2010-06-19

**Authors:** Ingeborg L. Zijdenbos, Margriet C. de Haan, Gerlof D. Valk, Olle Th.J. ten Cate

**Affiliations:** 1 University Medical Center Utrecht, The Netherlands; 2 Academic Medical Center Amsterdam, The Netherlands

**Keywords:** **Keywords:** Peer teaching, near-peer teaching, peer-assisted learning, clinical reasoning, case-based learning, undergraduate medical education

## Abstract

**Objectives:**

There is growing evidence for the value of several forms of peer teaching in medical education. Little is known about the feasibility of such an approach in courses of clinical reasoning. The University Medical Center Utrecht offers a clinical reasoning course for first and second year students which previously had been occasionally led by sixth year, i.e. near-peer students. We evaluated a version of this course, fully delivered by final year students.

**Methods:**

In 2008-2009 this highly structured mandatory clinical reasoning course for second year medical students was fully tutored by final year medical students, as part of a teacher training course in their core curriculum. Routine evaluations before and after introducing near-peers as tutors were compared, a focused questionnaire survey was conducted, as well as an interview with a group of students to evaluate the new format.

**Results:**

There was no difference in the ratings of the course before and after the introduction of the new format. In general, second year students are satisfied with the near-peer teachers. Strong points mentioned are their high motivation, involvement, enthusiasm, adjustment of cognitive level of teaching to the recipient students and stimulating skills.

**Conclusions:**

Although our study cannot provide evidence for differential learning effects, the evaluation of our final year student led clinical reasoning course shows encouraging results.

## Introduction

Growing theoretical insights and empirical evidence support the value of peer teaching in medical education.^1^^-^^7^ Several studies have shown that senior students are able to teach junior students without compromising achievement.^8^^ -^^15^ Most of them focus on clinical or practical skills ^13^^,^^14^^,^^16^ but peer teaching is also feasible for other objectives of education.^8^^-^^11^ Until now, little is known about the feasibility of peer teaching in clinical reasoning courses. One might doubt whether medical students can be employed in teaching clinical reasoning. This typically appears to be the domain of experienced clinicians, showing from practice how physicians think and decide. However, if teaching materials are highly structured the learning effects are less dependent on teachers’ expertise.^17^^,^^18^ That would mean that a highly structured clinical reasoning course could potentially be delivered by near peers. Peer teaching is usually confined to teaching among students of equal level, while near-peer teaching refers to the teaching of students by higher year students. The Utrecht University Medical Centre offers highly structured courses in case-based clinical reasoning for first and second year medical students since 2002. Until 2008, sixth year students (“near-peer teachers”) have occasionally assumed teacher roles in these groups and were usually favourably evaluated. Based on these positive experiences we decided to let all clinical reasoning sessions in the second year in 2008-2009 be guided by final year near-peer teachers. This paper elaborates on our experience with this student-led course.

### Methods

A Case Based Clinical Reasoning (CBCR) course for first and second year students is part of the core curriculum, aimed at practicing clinical problem solving skills and initial development of illness scripts. The course, 17 sessions of 2 hours, spread over the first and the second year program, is based on written clinical cases, starting with a clinical presentation including symptoms, complaints or other characteristics of an individual patient. The cases refer to clinical problems of which the underlying patho-physiology has been studied in previous block-courses.The second year CBCR course consists of 9 sessions of two hours over an eight-month time span (September-April). In every session, a clinical case is discussed in groups of twelve students. The groups stay together for all 9 sessions. Cases include clinical vignettes and questions that guide students through the clinical course. Most of the tutorial session is led in turn by two to three students from the group, with a consultant clinician present to guide the students when necessary. The student peer teachers receive additional information such as patient answers to history questions, physical examination findings, diagnostic findings, which they all reveal to the other students at the right moment during the session.^19^^,^^20^ At the end of the year, a written exam is administered to the students.During the CBCR course for first year students, every group has its own consultant clinician who facilitates nine sessions. In their second year, they now face sixth year “student consultant who replace the consultant clinicians. In this paper we will predominantly use the term ‘student consultant’ to signify the sixth year near-peer teachers who act as a tutor, because (a) ‘consultant’ is regular terminology used in CBCR courses and (b) to avoid confusion with the within-group student ‘peer teachers’ who largely lead the sessions. In another publication we have named this model ‘nested peer tutoring’, as supervision of within-group peer teaching is provided by near-peers.^21^The student-consultants teach a CBCR-session as part of an obligatory teacher training course in the final year of the curriculum. They are well-prepared for teaching as they have attended an educational training course and have received extensive instructions on the content of the case, related to the session they guide.^21^ Per year, about 250 sixth year students must each guide one CBCR session. As there are 24 groups in the second year cohort and each has 9 CBCR sessions, a total of 216 sessions per year must be guided by sixth year student-consultants. The teacher training course is delivered about 15 times a year during approximately the same months as the second year CBCR course. These sixth year students are on average 24-26 years old, with 60 to 70% females and 30 to 40% males.To provide the second year groups with a sense of continuity over sessions, every sixth year student-consultant fills out a report to be passed on to the next student-consultant of the particular group. The report contains remarks on group functioning, on individual students if necessary, on knowledge gaps to be discussed in a future session and on agreements on assignments or conduct made with the group. All sixth year student-consultants are provided with a sheet of photos and names of second year student group members.

### Ethical considerations

This format may raise ethical questions, as clinician-teachers are replaced by final year medical students. The Netherlands have no formal instrument to provide ethical approval for medical education research or medical curriculum innovations. Nevertheless, we had thorough deliberations before we decided to start this educational experiment.There were a number of considerations that made us believe that this curricular change could be made. In the past, the experience of clinical consultants ranged from subspecialists to recently graduated physicians. Based on program evaluations and previous studies, we had no reason to assume that inexperienced physicians were less well equipped to teach in such courses.^22^ Our experience with final year medical students in this CBCR course has been built over the past four years. In this period we occasionally had sixth year students take over such lessons. In no instances have we observed any adverse effects, compared to regular clinical teachers. Next, clinical consultants, responsible for a group throughout the year, have often voiced the problem that their specific content expertise did not fully fit with many of the various cases discussed over the year. In several cases their knowledge would be at best no better than that of medical students approaching graduation. For all sessions, detailed written documentation for students, within-group peer-teachers, and consultant teachers respectively is provided.The course is highly structured and as much as possible self-contained, as second year peer-students must largely run the course. This makes the dependence on the consultants limited. This format was introduced in 1992 and still works well.^18^ The level of this course, i.e. for second year medical students is such that we believe it can be well managed by nearly graduated medical students. Finally, we have theory-based reasons to believe that the teaching by senior medical students may even be beneficial in certain respects. This notion is derived from what has been called cognitive congruence and social congruence theory, which concepts maintain that a smaller academic distance between teachers and learners helps to match explanations with understanding and helps to improve social understanding in a way that enhance learning.^2^^,^^5^^,^^23^^-^^25^

### Evaluation

We evaluated the course in three different ways. First, we compared the routine quality monitoring of the course, using a student questionnaire, before and after introducing the near-peer teaching format on relevant items. Second, we conducted a focused questionnaire survey after five months, and third, we interviewed a sample of students.Results from the routine evaluations by students at the end of the year (response rates around 90%) did not show different ratings of the CBCR course over the two years (Table 1).

**Table 1:** Mean scores and Standard Deviations for relevant items from routine evaluations

Relevant item (and scoring range)	2007/2008 clinical consultant (N=260)	2008/2009 student consultant (N=249)
Average rating for the CBCR course (1-10)	6.9 (1.3)	7.2 (1.3)
Guidance by the consultant was stimulating (1-5)	3.7 (1.0)	3.8 (0.9)
The consultant was well aware of the goals and format of the session (1-5)	3.8 (1.0)	4.1 (0.7)
The consultant provided sufficient guidance and information (1-5)	3.8 (1.0)	3.9 (0.8)

A short online survey among second-year students about their perceptions of the student-consultant teaching, compared to previous clinical consultant teaching was filled out in January 2009, after five sessions, by 35% of the second year medical students (96 out of all 274 students approached). Both closed format questions (Table 2) and space for additional comments were provided.

Additional comments illustrated how some students liked their previous clinical consultant better, particularly those students who indicated that they had been very lucky with a superb clinical teacher. Other students indicated they were happier with the student-consultants.Of the 96 second-year students who filled out the online survey, 12 showed their willingness to participate in a subsequent group discussion by providing their email addresses. From 6 different CBCR-groups 6 students eventually attended this meeting. During the session students were asked to define strong and weak points of student-consultants and clinical consultants respectively, and in addition to clarify their preference for near-peers as tutors of the CBCR course if this was the case.The students indicated that consultant clinicians in general have more contextual knowledge and provide more details while explaining unclear subjects. As a weaker point, they mentioned that clinicians tend to interfere more rapidly in group discussions, sometimes hindering the learning and reasoning process.Among the advantages, high motivation, involvement and enthusiasm of student consultants were mentioned, and the fact that they acted more at the same cognitive level when giving explanations. One participant said: “*You can really see that the sixth-years use clinical reasoning skills like we practice them in the CBCR course to solve clinical problems. They still understand how we reason and why things go wrong in our reasoning. I think this is difficult for some physicians as they are so much further in their career and just ‘know’ a lot of things without realizing which reasoning process is behind it.”* Other advantages mentioned were a more thorough preparation, and eliciting more basic knowledge instead of specific knowledge. It was indicated that during discussions, a student-consultant decreases fear of failure in younger students. In addition, they tend to better stimulate discussions and ask more questions before they reveal content information. Two disadvantages of student-consultants were mentioned. First, they have less clinical experience and therefore give fewer practice examples. Second, students indicate that student consultants tend to be less demanding, as they often give higher marks for active participation than clinician-consultants.As the teacher training course is obligatory for every final-year student, we asked whether second-year students had noticed any negative consequences of this involuntary aspect, such as less motivated tutoring. None of the six students interviewed had noticed such effects. Again, explanations given were that the student-consultants seem highly motivated and have a large sense of responsibility, maybe because they have only one teaching experience in their teacher training course which most of them seem to highly appreciate.

**Table 2:** Views of a sample of second year students after 5 sessions (N=96)

Item	Clinical consultant	Student consultant	No difference
Which teacher stimulates the clinical reasoning process best?	33.7%	34.7%	31.6%
Which teacher has the best didactic skills?	31.6%	36.8%	31.6%
Which teacher has more knowledge about all topics?	44.2%	26.3%	29.5%
Which teacher has the most power?	41.5%	7.4%	51.1%
Who stimulates your own reasoning process best?	25.3%	42.1%	32.6%
Who made you learn the most?	28.4%	37.9%	33.7%
Which teacher do you prefer?	28.4%	42.1%	29.5%
The tutoring by the student consultant was stimulating (scale 1-5)			3.7 (sd 0.8)
The student consultant gave sufficient guidance and information (scale 1-5)			3.8 (sd 0.8)
The constant change of consultants gave no problems with continuity (scale 1-5)			3.8 (sd 1.2)

Concurring with the results of the questionnaire, the discussion group students’ opinions on the CBCR group continuity varied. Some experienced negative effects of rotating student-consultants; they indicated that the reasoning process and group dynamics development is better when a group has one tutor for all nine sessions. Other students did not consider this a problem; they stated that the variation of teachers enabled them to learn from several examples and methods. In addition, it diminished the risk of having a less motivated tutor during the entire academic year. They also stated that continuity is not such an issue for the CBCR course as there are only 9 sessions of 2 hours, distributed over a relatively large period of time (8 months).

### Discussion

The evaluation of the CBCR program with student consultants shows encouraging results. Second-year medical students seem generally satisfied with near-peers as teachers. Advantages of near-peer teachers mentioned include their motivation, their discussion stimulating skills and ability to adapt to the cognitive level of the students. A set-up with rotating tutors does not seem to cause hindrance of continuity and group functioning.Despite the quite extensive literature on clinical reasoning processes^26^, there is a paucity of information on teaching methods for very early clinical reasoning and illness script development. While clinical experience and an adequate knowledge base seem important for clinical reasoning, this can evidently not be expected from students who have left high school only one or two years before. The issue is then: when and how to start helping students to reason like clinicians? We believe that it would probably be most helpful to provide junior students with not too detailed clinical cases and to write them in such way that previously acquired patho-physiology knowledge must be applied. At the end of 9 sessions students should have acquired a rudimentary idea of nine common illness scripts: most common symptoms and history, etiology, diagnostic findings to be expected, most common treatment, follow-up and prognosis. The idea is that a number of such general scripts, even when learned by heart for an end-of-term examination, could provide a rough semantic network that allows for further tuning, accretion and restructuring of clinical knowledge in subsequent years of medical education.^27^ Facilitating this learning process may be better done by intermediates than by experts. Possessing expertise may not always be helpful in teaching novices, as experts often cannot articulate their own expertise well.^28^ A significant advantage of the transition we made is the opportunity offered to all sixth year medical student to serve as a teacher at least one time in their student lifetime, and be coached to do so.^21^^,^^29^The feasibility of this approach may be helped by our local circumstances, such as an existing well-structured CBCR course which is highly self-contained. Students largely run the sessions by themselves with focused instruction and coaching of the student-consultants. This teaching format has some features similar to problem-based-learning, small groups, tutoring instead of didactic teaching. Applying a near-peer teaching approach in PBL-like curricula should seem feasible too.^11^^,^^23^While efficiency considerations were not the primary motive for shifting from a clinician-based to student-based CBCR course, the teaching load of our second year CBCR-course is now largely accounted for by student consultants, which in fact indeed diminishes costs. However, coordination tasks take relatively more time, because of the complicated logistics with sixth and second year timetables, the instruction of student consultants and the need for high quality materials.One limitation of our study is the lack of evidence on differential learning effects in students. As a different type of end of course examination was applied, a comparison between students’ achievements with clinical and student consultants in the previous year was not possible. Our evaluation is therefore limited to the views of students. Another limitation is a low response rate (35%) for the focused questionnaire survey, but as it concords with the routine evaluation results, we feel that it does reflect what the second year students generally think. What we have lost by this transition is a role modelling opportunity for experienced clinicians, but we do not have the impression that this drawback would outweigh the benefits of this student-led course.Ongoing monitoring and investigating differential learning effects will be necessary to further substantiate these positive results, but the first year of the clinical reasoning course with near-peer teachers proved to be a positive experience for both sixth-year and second-year students.
